# Barriers to Nurse-Patient Communication in Cardiac Surgery Wards: A Qualitative Study

**DOI:** 10.5539/gjhs.v6n6p234

**Published:** 2014-08-15

**Authors:** Vida Shafipour, Eesa Mohammad, Fazlollah Ahmadi

**Affiliations:** 1 Department of Medical-Surgical Nursing, School of Nasibeh Nursing and Midwifery, Mazandaran University of Medical Science, Sari, Iran; 2 PhD Graduated in Tarbiat Modares University, Tehran, Iran; 3 Department of Nursing, Faculty of Medical Sciences, Tarbiat Modares University, Tehran, Iran

**Keywords:** communication barriers, qualitative content analysis, nursing, cardiac surgery, patient experience

## Abstract

**Background:**

An appropriate and effective nurse-patient communication is of the most important aspect of caring. The formation and continuation of such a relationship depends on various factors such as the conditions and context of communication and a mutual understanding between the two. A review of the literature shows that little research is carried out on identification of such barriers in hospital wards between the patients and the healthcare staff.

**Objectives:**

The present study was therefore conducted to explore the experiences of nurses and patients on communication barriers in hospital cardiac surgery wards.

**Design and Methods:**

This qualitative research was carried out using a content analysis method (Graneheim & Lundman, 2004). The participants were selected by a purposeful sampling and consist of 10 nurses and 11 patients from the cardiac surgery wards of three teaching hospitals in Tehran, Iran. Data was gathered by unstructured interviews. All interviews were audio-taped and transcribed verbatim.

**Results:**

Findings were emerged in three main themes including job dissatisfaction (with the sub-themes of workload tension and decreased motivation), routine-centered care (with the sub-themes of habitual interventions, routinized and technical interventions, and objective supervision), and distrust in competency of nurses (with the sub-themes of cultural contrast, less responsible nurses, and their apathy towards the patients).

**Conclusions:**

Compared to other studies, our findings identified different types of communication barriers depending on the nursing settings. These findings can be used by the ward clinical nursing managers at cardiac surgery wards to improve the quality of nursing care.

## 1. Introduction

Effective and skillful communication is a crucial and an important element in the quality of nursing care (Fleischer et al., 2009). Employing effective communication skills as a valuable tool enables nurses to assess patients’ needs and provide them with the appropriate physical care, emotional support, knowledge transfer and exchange of information (Caris-Verhallen et al., 2004). High standards caring behaviors resulted from the appropriate nurse-patient communication will lead to health promotion and therefore patient satisfaction (Caris-Verhallen et al., 1999). Communication is a multidimensional, complex and dynamic process (Sheldon et al., 2006) and has been considered to be the most important part of a nursing routine job referred to since Florence Nightingale in the 19th century (Fleischer et al., 2009). Although the theorists (Watson, Travelbee, Paterson and Zderad) have dealt with the importance of nurse-patient relationships based on their own specific views (Hagerty & Patusky, 2003), however they all admitted that an appropriate relationship plays a key role in the provision and reception of healthcare (Sheldon et al., 2006).

To establish a proper skillful relationship with the patients, the nursing staff attitude should be changed to a patient-centered approach, a concept which is fully understood and accepted by the health authorities i.e. they pay respect to the patient’s autonomy, voice and decisions rather than merely delivering nursing services (College of Nurses of Ontario, 2006). Since nurse staff are responsible for the establishing and continuous improvement of this relationship (Bolster & Manias, 2010; Pronovost et al., 2003), therefore, the initial step in constructing such an effective communication should be to consider their needs and to fully support them (Manongi et al., 2009). In return, they will address the patients’ cultural, spiritual, mental, psychological, physical and social needs (College of Nurses of Ontario, 2006). Failure to communicate effectively is a major potential obstacle in the provision of delivering standard services in caring settings. This can result in anxiety, misunderstanding, misdiagnosis, possible maltreatment, exposure to complications, increased length of hospital stay, waste of resources and finally dissatisfaction of nurses and therefore possible misplacements as a result (Pronovost et al., 2003).

Despite strong emphasis on training and improving the caregiver’s communication skills, there are still obvious shortages and a good communication is restricted by a number of structured factors (Fleischer et al., 2009). Although in Iranian healthcare system compared to patients, nurses have more authority, role and power, the intensive care nursing staff still experience having problem to establish effective communication with the critically ill patients despite employing high levels of knowledge, experience and communication skills (Llenore & Ogle, 1999). The way ahead to resolve this failure depends on the situation and the causes of the problem (inadequate time, sensitive conditions and urgency of the patient) and the interactions of the carers and patients. Such interactions between carers and patients are varied and may need different approaches depending on the cultural values, level of experience and individuals’ personalities (Sheldon et al., 2006).

Cioffi (2003) believes that communication is more difficult when patients and carers cultural values and languages are different. Such a language barrier is causing inability in exchange of information and therefore a potential for misdiagnosis and maltreatment, especially in the case of patients with acute conditions. Moreover, this results in increased workload and dissatisfaction of the caring staff and possible more communication problems (David & Rhee, 1998; Garra et al., 2010), though negligence and lack of support of the nurses’ should not be ignored and must be addressed by the healthcare authorities (Manongi, et al., 2009). Therefore, recognition of communication barriers is the first step in improving nurse-patient communication (David & Rhee, 1998).

The relevant research in Iran is carried out by a descriptive and a quantitative approach with no focus at cardiac surgery patients. Previous research is also not free of bias and has evaluated nurse-patient communication barriers according to the predetermined criteria (Anoosheh et al., 2009). Apart from disease specific conditions, understanding the true experiences and feelings of the patients and nurses in the specified clinical field requires conducting qualitative research (Krippendorff, 2012). Compared to other wards, the cardiac surgery are spending much of their time with the patients in different situations such as pre- and post-operation (unconscious intubation, conscious intubation, and without intubation) and the way these nurses communicate with the patients is different from that in other clinical settings. Therefore, it is highly important to understand how this relationship develops.

The present study was therefore carried out to explore in detail problems associated with effective communication between patients and the caregivers in the cardiac surgery units.

## 2. Methods

The present research used a qualitative content analysis approach (a scientific method that leads to promoting the researcher’s understanding and knowledge of different phenomena and to express the emotional responses and the potential meanings in individuals’ daily lives, (Krippendorff, 2012) aimed to explore the cardiac surgery units participants’ experience of bilateral communication and its barriers.

### 2.1 Setting and Participants

Different ethnicities live in Iran each with their own specific culture, language and dialect. The setting used for this study included the central, highly referred and specialized hospitals in the field of cardiovascular surgery in Tehran, Iran. A large number of patients with various ethnicities from different parts of the country are referred to these hospitals on a daily basis. Therefore, the way the nurses interact with patients and the communication barriers encounter are different, complex and, sometimes, unpredictable. The wide variation patients and the nursing staff belief, cultures, languages and regional dialects may potentially limit effective interaction and communication, The people (either patients or their relatives) referred to these centers are more interested in speaking in their own local language (e.g. Azeri, Lori, Kurdi or Arabic), some of them even unable to speak in Persian, the country recognized formal language. Such a difference in language between nurses and patients often result in lack of interaction, misunderstanding and therefore, considered to be an important barrier for effective communication.

The research setting included the cardiac surgery wards in three teaching hospitals in Tehran, Iran. Sampling was purposeful and incorporated 21 participants including 11 patients (7 males and 4 females) and 10 nurses (2 males and 8 females). The inclusion criteria were: permanent employment of the registered nurses working in the intensive care unit of cardiac surgery and the patient candidates for surgery or the patients who had undergone surgery. Awareness of the phenomenon under study, vigilance, interest and ability to communicate in Persian language were of the common criteria for both the nurses and the patients (Table 1).

**Table 1:** Characteristics of patients and nurses (n=21)

Variable		Patient	Nurse
Gender	Male	7	2
	Female	4	8
Age (years)		49-65(51)	26-50 (66)
Ethnicity	Persian	4	7
	Azeri	5	3
	Arabic	1	
	Other	1	
Education	Illiterate	4	
	Elementary school	4	
	High school	2	
	Bachelor’s degree	1	10
Clinical diagnosis of cardiac surgery	CABG^a^	6	
	AVR^b^	3	
	Both	2	
Length of hospital stay (days)		7-28	
Work experience (years)			2-29

a Coronary Artery Bypass Graft

b Aortic valve replacement

### 2.2 Data Collection

The required data were collected through unstructured interviews from January 2013 to October 2013. The interviews with nursing staff were carried out individually in their break time and with the patients by their bedside.

In order to remove the ambiguities and to fully explore and clarify all aspects of problem, an in-depth interview was carried out individually. Therefore, the sampling continued to the level of data saturation. Totally, 26 interviews were carried out with 21 participants. The interviews lasted between 30 to 120 min, began with general questions from the nurses, such as: “Please describe the way you communicate with a patient on a routine day.”, and from the patients, such as: “Please explain how you interact with the nurse and how the nurse interacts with you”. Then it was continued with clarifying and probing questions such as: “Explain it more; what does it mean?”, “Can you give me an example so that I can understand your feelings better?”, and some more leading questions about the factors involved in nurse-patient communication problems.

### 2.3 Data Analysis

Data analysis was carried out based on the steps suggested by Graneheim and Lundman (2004): To gain a whole cognition, initially, the audio taped interviews were transcribed. The entire interviews were coded as the unit of analysis; words, sentences or paragraphs of the texts were regarded as meaning units. Then the relevant meaning units with regard to the main content were placed close to each other and were coded by a label. Reviewing of the whole text coding, comparison of the codes based on their similarities and differences, and their categorization into sub-categories were performed via a more abstract label. Through careful and deep reflection on the initial categories, the researchers’ agreement on the categorization of the codes, categories and sub-categories, comparison of the categories with each other and the hidden contents of the categories were expressed eventually in the form of the study themes.

### 2.4 Consideration of Rigor

Through method triangulation (interview and field notes) and time triangulation (interviewing the nurses working in different shifts i.e. morning, evening and night shifts) along with sampling matched to shift variations, that is selecting and interviewing patients from different background and nurses (in terms of individual characteristics in each group), the credibility and the transferability of the data were increased. The accuracy of coding and categorizations of the interview text was investigated by the fellow researchers.

In addition, the confirmability was undertaken by the peer checkers (through interviews and their relevant codes) and for the audibility, all of the study steps were detailed reported.

### 2.5 Ethical Considerations

Approval to conduct this study was granted by the Research Council and Ethics Committee of Tarbiat Modares University (Tehran, Iran), and the authorities of the mentioned hospitals was collected before data collection was carried out. After the approval of the proposal in the Research Council of the Medical Sciences College was granted, the study was described to potential participants and the participants who agreed to contribute in the study completed a consent form for interviewing and audio-taping the content of the interviews. They were also given flexibility to choose a convenient place and time for the interview.

The research ethical principles such as conscious content, anonymity, privacy and the participants’ will to withdraw from the study were observed.

## 3. Results

**Figure 1:**
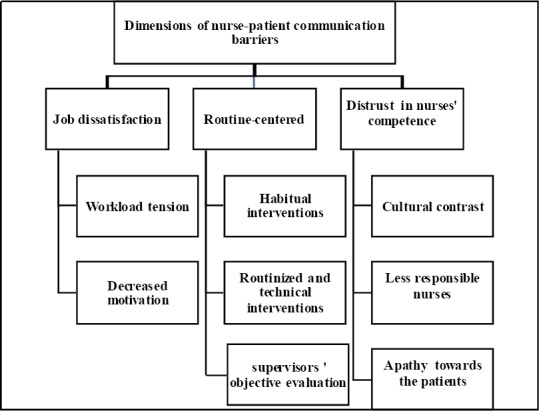
Dimensions of nurse-patient communication barriers

The data analysis emerged 670 codes, 16 sub-categories, 8 categories, and 3 themes including: “job dissatisfaction”, “routine-centered”, and “distrust in the nurses’ competence”, which were preventive in the process of nurse–patient communication. Each theme has been explained along with the relevant categories in Figure 1.

### 3.1 Job Dissatisfaction

Job dissatisfaction was the most predominant barrier which was mentioned by the nurses for effective communication with the patients. Workload tension and decreased motivation are two attributes in the theme of job dissatisfaction.

The workload and working under pressure, challenging nursing tasks, shortage of highly skillful nurses, and challenges approaching the patients’ relatives contribute to the nurses’ workload tension, which distract the nurses’ attention from the patients’ needs. The high volume of paper work, frequent patient admissions and discharges, imposed long shifts, working under stress, continuous care of infectious patients with surgical complications or abusive addicted patients resulted in increasing nursing workload, conflict and tension, which in turn led to increased dissatisfaction amongst the nursing staff and therefore contributed to lack of effective communication with patients: *“Intensive care is stressful and tiresome; there is a high degree of workload. If there is a critically ill patient or an agitated patient, or if there is a code for that, the work pressure gets so high that remains no possibility of speaking with the patients” (Nurse 2, Female)*.

Shortage of competent experienced nursing staff and the high patient–nurse ratios increases work related stress and work pressure and hinder bilateral communication between staff and patients: *“30 patients are assigned to 2 nurses; if one of these nurses is inexperienced, then the other gets burnt out” (Nurse 5, Female)*.

Another major identified problem was the presence of too many patients’ relatives as visitors timelessly and their interference with the care and treatment of patients. This has created more complications, delayed in patient’s recovery, took the nursing staff time tired and disturb the nurse–patient communication: *“The relatives worsen the nursing task; after the operation, the patient should stand up and walk so that he/she can breathe better, and the lung get cleaned. They don’t allow the patient to move; this may result in the patient’s cardiac tamponade” (Nurse 9, Female)*.

Inappropriate team position and low and disproportionate income to this highly demanding position are the attributes of decreased nursing staff motivation. In spite of the relentless efforts and care the nursing staff offer to patients, they are usually facing lack of respect and abusive behavior verbally or physically by the patients’ relatives; which is another contributory factor for lack of motivation: *“You save the patient, and then his/her relative arrives, when you say: Visiting is impossible at this time, they swear at you or grapple with you” (Nurse 8, Male)*.

Lack of appreciation and job promotion as well as the authorities’ lack of attention and ignorance towards the nurses’ efforts has affected the motivation of staff, which may adversely affect their level of standard care and communication with the patients: *“When there is no promotion, you get discouraged” (Nurse 3, Female)*. The system’s lack of support, appreciation and ignorance to meet the required resources nursing staff needed are other contributory factors for their lack of motivation: *“When nothing is done to resolve our problems, there remains no motivation for communication” (Nurse 7, Male)*. Moreover, the nurses did not consider their income proportionate to their workload and deemed less than standard level for meeting their needs. Thus they associated this with lack of motivation to offer the best practice and to effectively communicate with patients: *“You are awake overnight; you work on holidays but you don’t receive good salary. It is as if I work for free…I become impatient and grouchy, I don’t like to speak with the patients” (Nurse10, Female)*.

### 3.2 Routine-Centered

Habitual interventions and technical tasks in the form of a specified routine pattern are a reflection of the nurses’ job dissatisfaction and the nurse supervisors’ objective is to appraise their performance. Most nurses in the cardiac surgery setting prefer to concentrate on offering technical works, caring and meeting the patients’ clinical needs rather than focusing on the quality of their communication with patients. Therefore, communication based on spiritual and mental needs of the patients are at the lowest priority for the nurses: “*When the patient comes out of the operation room, he/she is intubated and goes under ventilator. Our entire attention is concentrated on weaning him/her gradually so that he/she is extruded” (Nurse 6, Female)*.

Technical nursing interventions (pre- and post-operation) had a prescriptive and authoritative state and implied a sense of inattention in the patients. They considered nurses the absolute agents of the physicians: “*The nurses do whatever the physician tells them; they don’t pay attention to anything else” (Patient 1, Male)*.

In their hospitalization period, patients receive a series of messages focused on mechanical and technical aspects of treatment, with no spiritual and psychological support. *“Our communication with the nurse is merely focused on taking medication and nothing more; they do not ask about my worries” (Patient 4, Female)*. Moreover, non-trasparent appraisal carried out by the nursing managers or supervisors and fear from being impeached and the accountability for following the pre-set task by their seniors were factors for performing the routine tasks rather than patient’s needs or communication: *“What is important here is doing some routine and objective tasks such as setting ventilator, changing the dressing, etc. I am not going to be reprimanded for not speaking to the patients” (Nurse 1, Female)*. Although the nurses tried to support the patients as much as possible, apart from doing their nursing tasks, this benefit was mostly limited to the necessary clinical care rather than spiritual needs: *“Atropine is injected but you can’t sit beside the patient to listen to his/her worries and to communicate to his/her about what he/she doesn’t know” (Nurse 4, Female)*.

### 3.3 Distrust in the Nurses’ Competence

Cultural conflicts, incompetent and less responsible nurses, and apathy towards the patients are the attributes of this theme. In the present study, cultural conflict, languages and dialects barriers along with difference in ethnicity were found as a cause of misunderstanding between nurses and patients: *“Say, the patient is an Azeri individual* *and I don’t understand his/her words, these all hinder easy communication” (Nurse 1, Female)*.

Also, because of gender difference, some male patients refrained from interaction and expression of their concerns with the female nurses: *“I can’t talk about my worries, the nurse is a female, and she doesn’t understand me” (Patient 11, Male)*.

Such beliefs resulted in male patients ignoring the advice of female nurses: *“Some male patients don’t listen to you; they feel disdained if a female nurse tells them what to do; they don’t accept it at all and here the problem arises” (Nurse 5, Female)*. Lack of responsibility by nurses was another attribute that was identified, from the nurse lack of attention, negligence and delay in fulfilling the patients’ educational, clinical and mental needs and was a factor in the distance of the patient from the nurse:

*“They just give the drugs and go; there comes no explanation with it” (Patient 2, Male)*.

Due to ignorance and shortage of time, the nurses failed to explain and keep the patients informed of their progress, especially in the case of surgery, which increased the sense of staff ignoring the patients: *“I was at the door of the operation room for three days; the operation was cancelled over and over, and they didn’t explain why?” (Patient 7, Male)*. The patients wanted to obtain some information about their clinical condition, operation/treatment plans and the post-operative care. The nurses failed to respond to the patients’ questions and referred them to the physician. This has created a feeling of the nurses’ incompetency and was regarded as a communication barrier:

*“Whatever you ask, they say: Ask your physician, or Hold it for later” (Patient 6, Female)*. In case patients not receiving the necessary information from the nurses on a timely manner, they were considered to be incompetent and inexperienced, therefore patients focus on initiating communication with medics to find answers to their questions. This in turn creates a sense of distrust and causes poor nurse–patient communication: *“If I want an answer or explanation, I ask the physician, because the nurses know nothing, and they can’t answer my medical questions” (Patient 10, Male)*. Apathy towards the patients is another attribute of the distrust theme. By being hospitalized and being away from their families, the patients encounter severe emotional pressures such as fear from death, anxiety, depression, anger, and finally, the feeling of loneliness and anguish, thus they are willing to talk to sympathetic nurses to express and put away their concerns. The cold and apathetic behavior of the nurses and the absence of a sympathetic nurse by the patient’s bed were also considered to be barriers for effective communication: *“Their behavior is so cold that you can’t have an easy and close relationship with them” (Patient 8, Male)*.

Lack of attention, impatience, and harsh behavior of the nurses make the patients not willing to share and to talk to nurses; this implied distrust and was another communication barrier: *“Some nurses are impatient; they don’t speak to you, they give the drug and go out of the room quickly, and some of them behave such that you feel regretful of asking your question” (Patient 5, Female)*. When patients were neglected and not receiving the care they needed, they feel left alone with no respect. This feeling damage the trust between the two parties if any and resulted in “leaving me alone” feeling and limits communication: *“I have no relationship with the nurses; they feel no respect for the patient, they don’t explain to the patient and don’t do their job in time” (Patient 9, Male)*.

## 4. Discussions

The findings of this study showed that job dissatisfaction, routine-centered care and lack of trust to nurses are the major barriers between nurses and patients for achieving effective communication at cardiac surgery wards.

The most important finding of this study is that nurses’ inappropriate working conditions and workload affects directly or indirectly the quality of their communication and therefore minimizes their quality of nurses services to patients.

Workload tension and decreased motivation which are the two attributes of job dissatisfaction are also considered as communication barriers.

The overloaded staff, the difficult nature of the work and distress inherent in intensive care, shortage of highly skillful and competent nurses compared to the large number of patients, dispute with the patients’ relatives, and tiredness resulted from less relevant duties have preoccupied the nurses time and therefore created a heavily stressful environment so that they cannot establish a friendly trustful relationship with the patients.

Anoosheh et al. (2009) also pointed out that heavy workload, nurses’ difficult nature of duties, inadequate human resources and shortage of time are some of the communication barriers reported by Iranian nurses. It seems that a quantitative study look at pre-defined obstacles presents just some of the features of a sub-theme, not all of its dimensions, whereas qualitative research (such as the present work) reveals other unknown features as well.

In the present study nurses have allocated their duty time to only emergency and non- emergency activities so that, at the end of their shift, they felt that they have had no proper communication and contact with the patients. This was reported as the communication barrier. This finding is supported by Manias and Bolster (2010), who suggested that time constraint due to heavy workload and acutely unwell patients resulted in alteration of routine nursing care and the short interactions between nurses and patients in an acute hospital setting.

Almost full time presence of the patients’ relatives and interference with the process of patients’ care and treatment increases the staff workload, distract their focus on patients’ care and waste their energy. This will be getting worse once a disrespectful relationship is established unwantedly between the two parties. On the contrary, the study of Cioffi (2003) showed that nurses were employing families for achieving better communication and tackling the barriers in the case of unfamiliarity with the patients’ language and culture. Therefore, their presence was regarded as a facilitator of the communication.

Decreased motivation was another dimension of job dissatisfaction, which was considered as a barrier in the effective nurse-patient communication. Inappropriate and disproportionate position of nurses in the clinical management team as compared to their specialized endeavors and authority’s lack of attention to staff promotion, not recognizing and appreciating hard working staff along with low salary all resulted in the nurses’ lack of motivation in establishing a helpful effective communication with the patients.

Llenore and Ogle (1999) supported this finding and argued that ICU nurses avoid verbal interaction with ventilated patients because of work-related problems and lack of reward. Oshvandi et al. (2008) also regarded low income, violence, lack of facilities, absence of a clear and transparent job description, physician-centered culture in management teams, centralized management, and lack of authority as the factors involved in the decreased job motivation of Iran’s nurses.

Routine-centered job performance was the next theme of the communication barriers in the present study. In fact, the nurses concentrated on performing specialized, clinical and routine pre-set care activities in the pre- and post-operation stages. They are exclusively following the physicians’ recommendations with having no time to think about what they are doing. This damages their independency and therefore eliminates any chance of staff creativity. Their nursing care time was spent in providing routine patients’ critical/non-critical specialized care. This was more evident in the case of ventilator-dependent patients which resulted in keeping the nurses much more involved, busy and conscious, as a result neglecting the necessity of communicating with other patients.

Radtke et al. (2012) suggested that the priority of nursing staff in the medical and surgical ICU wards was to focus only on the patient’s clinical needs rather than initiating an effective communication. However, in the study of Magnus and Turkington (2006), it was reported that apart from medical barriers (decreased consciousness and intubation), which impact the effective communication, the nurses’ inability in lip reading, their lack of efforts to initiate communication, the patients’ painful time, depression and severe weakness were identified as major communication barriers in the ICU.

In the present study, sometimes, the nurses’ preoccupation in performing clinical duties was a barrier to communication. Llenore and Ogle (1999), pointed out that nurses in ICU are so focused on performing clinical duties that only 5% of their caring time is devoted to communication with the patients. Baillie (2005) however, regarded quick assessment of the patients’ as the most important communication barrier in the emergency unit. This appears to be inevitable given the nature of disease and the acute condition of patients. In the present study, the evaluation of nurses’ performance through subjective approaches by their managers had negative impact on the nurse-patient relationship.

Molazem et al. (2011) reported that the main focus of the Iranian surgery nurses was on performing routine clinical duties. Chant et al. (2002) argued those factors such as culture and the nature of work force the nurses towards being task-centered rather than multi-task performers. In the present study, the patients recognized that nurses preferred performing their predetermined duties rather than communicating with them. McCabe (2004) argued that patients’ perception of the nurses’ behaviors is an indication of nurses’ concentration, concern and priority on performing their routine duties over talking and communicating with the patients.

Distrust to the nurses’ professional capabilities and competence is another theme of the communication barriers. Lack of awareness of the in-patients cultural values, local languages and dialects found in this study resulted in misunderstanding the patients’ needs and was considered to be a communication barrier.

However, Anoosheh et al. (2009) did not find language diversity as a barrier for an effective communication between nursea and patients in Iran. This finding may be explained by the fact that both groups were familiar with the local language used by the patients.

Nevertheless, according to Garra et al. (2010) the most commonly reported barrier for an effective communication between nurses and patients was language in the emergency setting.

Gender difference was also identified as a communication barrier in the present study. The influence of the patient’s gender on the nurse-patient communication and on the patients’ decision to actively participate in the treatment process was different based on the cultural settings and beliefs.

Accordingly, due to their cultural and religious beliefs, most Iranian male patients prefer male nurses and avoid female staff contribution in management team, therefore not following advice made by the female staff, though in reality it is not always possible to provide male patients with matched male corers as male nurses constitute only 20% of the nurses’ population in Iran (Oshvandi et al., 2008).

The problem is becoming more obvious when a female nurse has to look after a male patient. This regarded as an impediment in the effective nurse-patient relationship. While Waldron et al. (2012) stated that in primary care, when both the providers and receivers of care service are males, the risk of refusing clinical advice and aid is increases. Therefore, the presence of at least one female nurse in the caring team will build more trust and fewer refusals. It seems that the gender match of care provider and care receiver is a disturbing factor in care. Lack of nurses’ attention, delay and carelessness in providing necessary information and fulfilling the patients’ care creates a sense of nurses’ lack of responsibility and therefore damages their reputation, which are regarded as communication barriers. Lotzkar and Bottorff (2001), reported that nurses’ behaviors such as clear, concise and straightforward communication for answering the patients’ questions on a timely manner along with taking appropriate actions is important in building a bilateral trust and demonstrating their competence.

Otte (1996) also delineated that weakness in communication skills or lack of communication resulted in lack of patients’ confidence to nurses. Attree (2001) suggested that problems in starting effective communication with patients resulted in incomplete information exchange, misunderstanding and increased patients’ concerns. In the present study, drug prescription which was performed on a routine basis by nurses deemed to be performed with no proper or the least verbal communication.

Caris-verhallen et al. (1999) indicated that paying too much attention in routine daily care leads to insufficient provision of care and information exchange to patients. However, Bolster and Manias findings (2010), indicated that most nurses have communicated with patients during the course of drug prescription.

In the present study, nurses’ lack of communication and referring patients to physician rather than addressing the patients’ requests/needs, created a sense of distrust to nurses’ knowledge and competency hence causing lack of respect for the important role they have in the caring team.

Therefore, the patients have received their information mainly from their physicians rather than from the nurses who are spending more time with them. Weber et al. (2007), however, reported that the verbal communication is mainly physician-focused though the patients received more information from the nurses. McKenzie (2002) also indicated that pregnant women have measured the care providers’ lack of attention, refusal, evasiveness and stalling in providing proper and on time response to the patients’ demand as barriers for effective communication. In addition, due to lack of expertise and experience, junior and less experienced nurses in this study avoided to be involved in caring for the more complex patients. Consequently, this resulted in a wrong interpretation of nurses incompetency and their lack of ’ knowledge. Llenore and Ogle (1999), pointed out that junior nurses avoided interactions with patients, and that the role staff experience is more essential in establishing verbal and effective communication than their theoretical education (Caris-Verhallen et al., 1999). Not allocating time by nurses to accompany patients who have already suffered from severe emotional pressures and the absence of establishing a bilateral friendly relationship with them deemed to create a sense of distrust to nurses’ capabilities. In such a situation, the patients do not empathy with the nurses and do not express their concerns. McCabe (2004) indicated that once nurses established such a closeness of feeling and empathy with the patients, then the patients would trust the nurses.

Expressing sympathy by the nurses can decrease the patients’ anxiety and worries. However, they feel disappointed and frustrated when encountering an apathetic relationship with the nurses. Treating patients with lack of respect and in aggressive non-friendly way can cause destruction of the built trust and inappropriate bilateral interactions. Such unfriendly (Park & Song, 2005) or aggressive (Anoosheh et al., 2009), behavior of nurses was reported by the patients as an inhibitory factor in establishing an effective communication. Although nurses attribute their inadequate communication with the patients to insufficient time and workload, task orientated caring system; traditional workplace policies and practices remain as barriers to communication and alter the quality of the care offered to patients (Chant et al., 2002).

## 5. Conclusions

Our findings showed that although the nature of the barriers in bilateral patient-nurse communications in hospital wards are similar in almost all countries, however, the attributes of barriers in the present study may differ; e.g. job dissatisfaction due to workload, uncontrolled presence of the patients’ relatives who may interfere with the nurses’ duties. Concerning distrust in nurses’ competency, which is rooted in masculinity, we may propose that it may be influenced by the participants’ cultural different values and as such, it may be another way of reflecting barriers. These findings may be useful for nursing managers in planning the caring processes accordingly such that they can provide a peaceful relaxing caring environment, minimize conflicts and therefore overcome the barriers, thus improving the quality of patients’ care. Furthermore, in order to improve the nurse–patient communication, more works to be done to provide a non-stressful trustful environment for both staff and patients that can be a basis of building bilateral trust, friendly relationship and effective communication. This will in turn prevent compromising the quality of care and services and therefore guarantees a high standard care services. Moreover, these findings may be used in developing a device that somehow assess and monitor the extend of communication barriers in cardiac surgery units.

### 5.1 Limitation

Since, the aim of qualitative research is not to generalize the findings; and the findings are dependent on the participants’ context and the study situation, the applicability of research findings is restricted, which can be considered as a limitation (for other practitioners).

Also, although the researchers tried to recruit more male nurse participants for the study, they were unsuccessful because of scheduling problems at the times of the interviews. This was also a convenience sample and, perhaps male nurses chose not to participate. The perception of communication barrier arising during nurse-patient communication in this study was that of more the female nurse participants. Whether the results would vary with more male nurses or in other groups of female nurses requires further investigation.
